# Matrix Metalloproteinases in *Helicobacter pylori*–Associated Gastritis and Gastric Cancer

**DOI:** 10.3390/ijms23031883

**Published:** 2022-02-08

**Authors:** Olga Sokolova, Michael Naumann

**Affiliations:** Institute of Experimental Internal Medicine, Medical Faculty, Otto von Guericke University, Leipziger Str. 44, 39120 Magdeburg, Germany

**Keywords:** inflammation, NF-κB, MAP kinases, type IV secretion system

## Abstract

Gastric cancer is one of the leading causes of the cancer-related mortality worldwide. The etiology of this disease is complex and involves genetic predisposition and environmental factors, including *Helicobacter pylori*. Infection of the stomach with *H. pylori* leads to gastritis and gastric atrophy, which can progress stepwise to gastric cancer. Matrix metalloproteinases (MMPs) actively participate in the pathology development. The further progression of gastric cancer seems to be less dependent on bacteria but of intra-tumor cell dynamics. Bioinformatics data confirmed an important role of the extracellular matrix constituents and specific MMPs in stomach carcinoma invasion and metastasis, and revised their potential as predictors of the disease outcome. In this review, we describe, in detail, the impact of MMPs in *H. pylori*–associated gastritis and gastric cancer.

## 1. Introduction

Normal and pathological proteolysis of the extracellular matrix and the processing of a variety of cell surface molecules are mediated by a big family of zinc-dependent proteases that consists of 24 MMPs (matrix metalloproteinases), about 35 ADAMs (a disintegrin and metalloproteinase) and 19 ADAMTS (ADAMs with thrombospondin domain) [1]. MMPs have a homologous structure represented by an N-terminal signal sequence, a catalytic site-interacting autoinhibitory propeptide and a zinc-containing catalytic domain. All MMPs, except for MMP-7, -23 and -26, have a hemopexin-like repeat domain next to the catalytic one, which serves for substrate binding. Most MMPs have a C-terminus regulatory domain. The MT-MMPs (membrane-type MMPs) possess, at the C-terminus, a transmembrane domain and a short cytoplasmic domain (MT1-MMP (MMP-14), MT2-MMP (MMP-15), MT3-MMP (MMP-16) and MT5-MMP (MMP-24)) or have a GPI (glycosylphosphatidylinositol) anchor (MT4-MMP (MMP-17) and MT6-MMP (MMP-25)). They also have the MT-Loop, an 8 or 9 amino acids insertion in their catalytic domain, which participates in furin recognition for their processing/activation [2,3]. Other MMPs are secreted and can exert intra- and extracellular functions. Based on their substrate preferences, MMPs were subdivided to collagenases (MMP-1, -8 and -13; main substrates include collagens I, II, III, V, etc.), gelatinases (MMP-2 and MMP-9; main substrates include collagens I and IV), stromelysins (MMP-3, -10 and -11; do not cleave collagen I, but II, IV and V), matrilysins (MMP-7 and MMP-26; do not cleave collagen I, but collagen IV), TM-MMPs and ungrouped (MMP-12, -19, -20, etc.) [4]. In addition to an intrinsic proteolytic activity toward classical ECM (extracellular matrix) molecules, e.g., collagens of different types, laminins, gelatin, vitronectin and fibronectin, activated TM-MMPs mediate processing of secreted MMP precursors, in particular, pro-MMP-2 and pro-MMP-13. Substrates of MMPs include ADAM9, ADAMTS4, CD44, ICAM-1 (intercellular adhesion molecule 1), syndecan 1, pro-TNF (tumor necrosis factor α), pro-TGF-β (transforming growth factor β), IL (interleukin)-8, dickkopf-1, E-cadherin and selected integrins [2,5]. Generally, MMPs have many common substrates within the ECM. Single but not double knockouts of different MMPs in mice did not disturb embryonic development, demonstrating a functional redundancy of MMPs. However, MMP knockouts have a number of life-threatening defects in, for example, bone development, wound healing, angiogenesis and innate immune response [6].

Localization of MMPs on the cell surface supposes their interaction with membrane protein complexes/receptors, which sense the environment. The lysis of the ECM by MMPs modulates focal adhesions and activates cellular signaling molecules, e.g., ERKs (extracellular signal-regulated kinases), which promote cellular migration and, in turn, impact MMP production [3,7]. Interestingly, several secreted MMPs, including MMP-2, -3 and -9, function intracellularly and can, for example, downregulate the activity of DNA repair enzymes PARP1 (poly(ADP-ribose) polymerase1) and XRCC1 (X-ray repair cross complementing 1), as well as NF-κB (nuclear factor kappa B) inhibitor IκBα (in response to oxidative stress). MMP-12 secreted predominantly by macrophages acts in the nucleus of nearby cells by suppressing IκBα gene expression [3,8].

MMPs activity is regulated (i) at the transcriptional level in a cell-type-dependent manner (in response to growth factors and cytokines); (ii) through proteolytic cleavage of their N-terminal signal peptide and prodomain (e.g., in MMP-1, -2, -7, -8, -9 and -13) by other MMPs or serine proteases (e.g., trypsin, plasmin and leukocyte elastase) in physiological conditions and in response to oxidative stress; (iii) via binding to TIMPs (tissue inhibitors of metalloproteinases) 1–4 [1,2,3]. For example, TIMP-2 links MT1-MMP with pro-MMP-2, and MMP-2 activation depends on the level of all complex components [2]. More detailed information about MMPs’ regulation in health and disease can be found in a number of comprehensive reviews [9,10,11].

*H. pylori* is a Gram-negative bacterium that colonizes the human stomach and causes acute and chronic gastritis. Chronic gastritis can progress sequentially to atrophic gastritis, when a loss of appropriate glands takes place; to intestinal metaplasia, when gastric mucosa further acquires intestinal features; to neoplastic stage without invasion, called dysplasia; and, finally, to gastric cancer [12,13]. A progression of gastric cancer might also be associated with *H. pylori* infection [14]. In a group of patients, infection with virulent *H. pylori* strains contributed to the development of gastric ulcer disease and MALT (mucosa-associated lymphoid tissue) lymphoma. It is believed that, in addition to the bacteria, host genetic susceptibility, salt diet and smoking increase the risk of carcinogenic transformation in human stomach. Among the genetic factors involved in gastric pathology, inherited or somatic mutations in genes encoding cell structure molecules (e.g., E-cadherin and adenomatous polyposis coli protein), as well as SNPs (single nucleotide polymorphisms) in genes encoding pro-inflammatory cytokines, including IL-1β, IL-8, TNF, some MMPs, TIMPs and defensins, have been described [15,16,17]. An epigenetic gene regulation in gastric tumorigenesis, especially through promoter methylation/demethylation and histone modifications, is also under intensive investigation [18].

**Figure 1:**
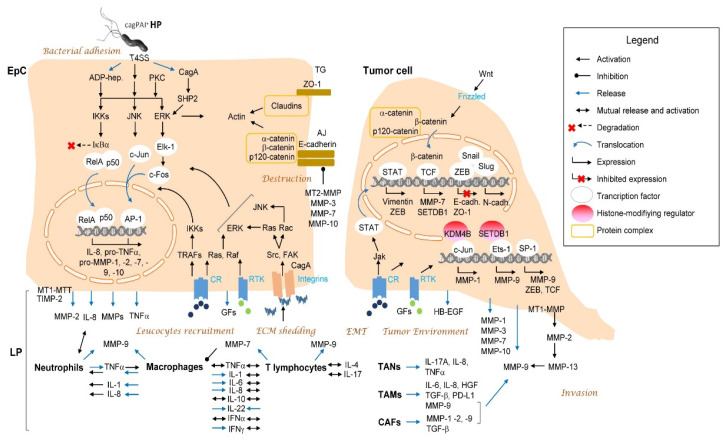
Signaling pathways pivotal for MMPs expression and MMPs-related processes in gastritis and gastric cancer. In tumor cell, the NF-κB, JNK and ERK MAPK pathways activated in response to *H. pylori* and cytokines are not shown for simplicity. The transcription factors and their regulators important for expression of the EMT-related genes and discussed in the review are depicted in the tumor cell. The immune cells are recruited to the tissue and release a big number of mediators, including MMPs. MMPs from different cells populations activate each other, participate in matrix remodeling, regulate focal adhesion complexes and shed proteins in cellular junctions. It leads to disturbances in the cellular contacts and restructures actin, which impacts the cellular motility. MMPs can also activate cytokines and growth factors embedded within the extracellular matrix. In the tumor microenvironment, both cancer cells and tumor-associated immune cells keep producing the growth factors and MMPs, supporting the motility and invasion of tumor cells. AJ, adherens junctions; GFs, growth factors; CR, cytokine receptor; ECM, extracellular matrix; EMT, epithelial–mesenchymal transition; EpC, epithelial cell, HP, *H. pylori*; LP, lamina propria; RTK, receptor tyrosine kinase; TGs, tight junctions.

For the successful colonization of gastric niche, *H. pylori* uses, among others, ammonia-generating urease, which compromises an acidic microenvironment; flagella, which mediate bacteria movement in the mucus glycoprotein layer; and outer membrane proteins BabA, SabA, AlpA/B and OipA (outer inflammatory protein A) for adhesion to the gastric epithelium. Pore-forming VacA (vacuolating cytotoxin A) and secreted protease HtrA (high temperature requirement A) destroy host cells and epithelial integrity, respectively [19,20]. VacA gene is polymorphic, and genotypes s1/m1 and s1/m2 are associated with greater epithelial damage and infiltration of neutrophils and lymphocytes to the antral gastric mucosa, in comparison to other allele combinations. It has been found that the *H. pylori* strains related to peptic ulcer and gastric cancer contained the s1/m1 and s1/m2 *vacA* variants. It is believed that toxic VacA contributes to gastric ulcer disease; a direct involvement of VacA to gastric carcinogenesis is unconfirmed [21]. Moreover, cagPAI (cag pathogenicity island), a set of about 27–31 genes, and cagPAI-encoded bacterial T4SS (type IV secretion system) are the main virulence factors responsible for the activation of pro-inflammatory and pro-survival pathways in gastric mucosa, including the NF-κB and MAPKs (mitogen-activated protein kinases) [22,23,24] (Figure 1). The T4SS delivers a cagPAI product—CagA (cytotoxin-associated gene A)—to the host cell [25]. In the host cell cytoplasm, CagA becomes tyrosine phosphorylated within its EPIYA motifs mainly through the Src-family protein kinases. It leads to the activation of SHP-2 (Src homology region 2 domain-containing phosphatase-2) and to actin cytoskeleton rearrangements detected as “hummingbird” phenotype in cultured cells. Overexpressed in mice, CagA has oncogenic properties per se. In combination with other inflammation-associated virulence factors, CagA essentially contributes to severe gastric pathology [26]. CagPAI- and CagA-expressing *H. pylori* strains are strongly associated with gastritis and gastric adenocarcinoma development [27]. The intermediates of the lipopolysaccharide biosynthesis HBP (heptose 1,7-bisphosphate) and its more abundant and potent derivative, ADP-glycero-β-D-manno-heptose (ADP-heptose), are involved in the activation of NF-κB [28,29,30]. NF-κB regulates the transcription of many genes, especially of these encoding attractants for neutrophils, macrophages, T cells, IL-6, IL-8, TNF, CCL20 (CC-chemokine ligand 20), COX-2 (cyclooxygenase-2), Sonic Hedgehog, growth factors and some MMPs (described in following sections) [31,32,33]. *H. pylori*–induced MMPs production leads to modifications in the ECM and, thereby, influences the adhesion-regulated cellular signaling, including FAK (focal adhesion kinase), Src and Grb2 (growth factor receptor-bound protein 2). MMPs can contribute to chronic inflammation by promoting mucosal damage and facilitating interaction of the epithelium with bacteria, immune and stroma cells [34].

In this review, we summarize and briefly discuss the role of MMPs in pathogenesis of *H. pylori*–related gastritis and gastric cancer.

## 2. MMPs and Gastritis

### 2.1. MMP Levels and Localization

Infection with *H. pylori* causes inflammation of the stomach, which is characterized by a massive recruitment of immune cells, in particular, polymorphonuclear leucocytes and macrophages, to the gastric mucosa [35]. MMPs impact the inflammatory milieu and architectural changes in the infected tissue [36,37]. Many data, sometimes discrepant, exist concerning the upregulation of individual members of the MMP group in *H. pylori*–associated gastritis.

MMP-2, -7 and -9 are the most often investigated MMPs in the context of *H. pylori*–associated gastric inflammation by using a range of methods, including IHC (immunohistochemical analysis), RT-PCR (real-time polymerase chain reaction), zymography and ELISA (enzyme-linked immunosorbent assay). It has been shown that MMP-2, -7 and -9, as well as MT1-MMP and TIMP-2 and -4, were upregulated in gastric mucosa biopsies from individuals with *H. pylori*–associated gastritis. Flow cytometry revealed that these MMPs and TIMPs were increased on the surface of infiltrative mucosal lymphocytes rather than on epithelial cells, proportionally to the disease severity [38]. Strongly increased expression and activity of MMP-9 (and slightly increased of MMP-2), but no differences in TIMP-1 and -2, were detected in the biopsies of *H. pylori*–positive subjects in comparison to uninfected individuals [39,40]. Incubated for 24 h in cell culture medium, gastric mucosal biopsies from *H. pylori*–positive patients with chronic superficial gastritis produced higher levels of MMP-9 and TIMP-1, in comparison with biopsies from *H. pylori*–negative individuals [41]. Treatment with antibiotics and the proton pump inhibitors led to decreased levels of total and active MMP-9 in the gastric mucosa of both the antrum and the corpus parts in *H. pylori*–associated gastritis patients [42].

An increase of MMP-7 levels in gastric biopsy specimens from patients infected with *H. pylori* has been demonstrated by several studies [43,44,45,46]. In contrast to MMP-9, MMP-7 localizes preferentially on the surface of epithelial cells and deep in the gastric gland, where chief cells are distributed [43]. Studying the antrum- and corpus-derived *H. pylori*–positive biopsy specimens, Bebb et al. [44] have found an increased production of MMP-7 in the human epithelium, especially in the proliferative zone, and in immune cells. In cultured gastric gland cells, MMP-7 was localized in the advancing edge of migrating cell colonies. The downregulation of MMP-7 with antisense oligonucleotides led to decreased motility within the isolated gastric glands from *H. pylori*–infected patients [43].

It has been suggested that MMP-7 upregulation is linked to *H. pylori*–induced hypergastrinemia. In particular, an increased abundance of MMP-7 has been found in gastric corpus biopsies from hypergastrinemic patients [47]. Culture medium from gastrin-treated human gastric epithelial cells stimulated the proliferation of stroma myofibroblasts in a MMP-7-dependent manner via the MAPKs and PI3K (phosphatidylinositol 3-kinase) pathways [47]. Yin et al. [48] have demonstrated using IHC that neutralization of gastrin in the transgenic gastrin overexpressing INS–GAS (insulin–gastrin) mice led to a reduced expression of MMP-7 and proteins involved in EMT (epithelial-to-mesenchymal transition), including soluble HB-EGF (heparin-binding EGF (epidermal growth factor)-like growth factor) and Snail (Snail family transcriptional repressor 1). In gastric cell lines AGS, MGLVA1 and ST16 infected with virulent *H. pylori*, depletion of gastrin attenuated MMP-7 mRNA and protein production [48]. Furthermore, the inhibition of gastrin or MMP-7 in these cell cultures diminished the expression and shedding of HB-EGF and downregulated gene expression of the Snail, Slug (Snail family transcriptional repressor 2) and vimentin [48]. Thus, these in vitro experiments suggest that gastrin-induced MMP-7 enhances EMT in epithelium and stimulates stroma cells in the gastric mucosa, which might impact pathology development. However, another group has demonstrated the protective role of MMP-7 in *H. pylori*–related gastritis (and carcinogenesis) in vivo [49]. It has been observed that infection with *H. pylori* led to enhanced inflammation and cell turnover in gastric epithelium of MMP-7 (−/−) mice, compared with uninfected animals or with infected wild-type C57BL/6 mice. Similarly, enhanced inflammation and accelerated development of hyperplasia and dysplasia were seen in the MMP-7 (−/−) INS-GAS mice [36]. It is not clear whether MMP-7 could promote, for example, bactericidal activity protecting gastric mucosa, as it happens in the gut epithelium, where MMP-7 from the Paneth cells processes pro-defensins to their active forms [50]. Knockout of MMP-7 could be also compensated by other MMPs or impact on their networking. Anyway, macrophages from MMP-7 (−/−) mice expressed higher levels of IL-1β than those from the wild-type mice. It has been suggested that MMP-7 could be protective by suppressing M1 macrophage polarization in gastric mucosa [36].

Systemic levels of MMPs in infected persons have also been investigated. In serum of *H. pylori*–infected children, TIMP-1, but not MMP-2, -7, -8 and -9, levels were increased in comparison to the uninfected group [51]. In adults with *H. pylori*–related gastritis, increased levels of MMP-8 and -9, an unchanged level of MMP-7 and decreased levels of MMP-2 and TIMP-1 in comparison to healthy volunteers were detected in serum by using ELISA [52]. The neutrophil degranulation products neutrophil elastase and myeloperoxidase, which are the markers of the proteolysis and oxidative stress, were also increased in serum of the adults with *H. pylori*–associated gastritis [52]. An investigation by Yeh et al. [53] compared the serum levels of MMP-9, as well as MMP-3 and MMP-7, between *H. pylori*–infected patients and *H. pylori*–negative patients within the gastritis group, and no difference was found. Perhaps, detectable levels of these MMPs in serum reflect any established gastric inflammation. Furthermore, MMP-3 and MMP-7 were strongly elevated in the serum of patients with *H. pylori*–positive gastric cancer in comparison to the both gastritis groups [53]. Thus, concomitantly increased serum levels of MMP-3 and MMP-7 seem to be more relevant for gastric dysplasia and gastric cancer [54].

### 2.2. Bacterial Factors and Host Pathways Pivotal for MMP Activation

Among bacterial factors responsible for MMPs synthesis and secretion in inflamed gastric mucosa, cagPAI but not VacA has been described in in vivo investigations [46]. In particular, Wu et al. [55] have demonstrated that MMP-1 mRNA was overexpressed in samples from gastritis patients infected with cagPAI- and OipA-positive *H. pylori*.

To determine specific *H. pylori* effectors and host-cell signaling pathways responsible for MMPs production and activity, *H. pylori* wild type and mutated strains were co-cultured with gastric cell cultures (predominantly human cancer cells, including MKN7, MKN45 and AGS). In the cell lines MKN45, AGS and SNU638, as well as in primary gastric cells, infection with cagPAI- and OipA-positive *H. pylori* led to expression of the MMP-1 gene, as well as genes encoding for its promoter regulators c-Fos, c-Jun and PEA3 in a JNK (c-Jun N-terminal kinase)- and ERK MAPKs-dependent manner [55]. Pillinger et al. [56] confirmed an involvement of ERK in MMP-1 production in AGS cells in response to both CagA-expressing and CagA-negative *H. pylori* strains. However, CagA has been suggested to augment the MMP-1 secretion [56]. MMP-1 expression in P1 strain-infected AGS cells can be exerted through protein kinase C-dependent c-Fos expression and consequent activation of AP-1 transcriptional complex in a CagA-independent manner [24] (Figure 1).

It has been reported that *H. pylori* stimulates the expression of MMP-7 in a number of cell lines [43,44,57]. Among investigated *H. pylori* virulence factors, cagPAI and CagE, but not CagA and VacA, were important for MMP-7 production in vivo and in vitro [44,45,57,58]. Concerning a molecular mechanism, the MMP-7 expression was upregulated in AGS cells via Rho- and Rac-activated NF-κB and Rac-activated AP-1 [43]. In addition, a cagPAI-expressing *H. pylori* isolate 7.13 induced MMP-7 transcription in MKN45 cells via provoking the nuclear translocation of p120, which relieves Kaiso-mediated transcriptional repression of the MMP-7 gene. Similar results were obtained in the co-culture of isolated mouse gastric cell colonies and *H. pylori* SS1 strain [58].

Expression of MMP-9 in gastric cell lines was a result of cagPAI-dependent activation of NF-κB [39]. *H. pylori* induced AGS cell invasion through collagen type I and Matrigel via MMP-2 and MMP-9 in a T4SS- and c-Met-dependent but VacA-independent manner [59]. Of note, increased activities of MMP-1, MMP-3 and MMP-7 have also been implicated in the transmigration of infected cells through collagen and Matrigel matrixes [24,60].

The stimulatory role of CagA in MMP-3 and MMP-10 expression in AGS cells has been suggested [61,62]. In gastric biopsies and in cell culture, the expression of MMP-10 has been dependent on CagA status, and EGF receptor and Src activations. The inhibition of ERK and JNK MAPKs (but not p38) attenuated *H. pylori*–induced MMP-10 expression [62].

### 2.3. Cytokines and MMPs

Since MMPs’ expression is mostly regulated through the MAPK and NF-κB signaling pathways, which are targets for cytokines, a relation between cytokines and MMPs has been studied (Figure 1). Gastric biopsies from *H. pylori*–infected patients had higher levels of IL-1β, IL-8 and MMP-3 (by ELISA) in comparison to *H. pylori*–negative controls. The level of IL-1β positively correlated with IL-8 level, and both cytokines positively correlated with MMP-3. They were the highest in atrophic gastritis, compared with the non-atrophic gastritis and ulcer disease groups and increased in parallel to the atrophic grade. *H. pylori* eradication with antibiotics and a treatment with proton-pump inhibitor led to the decrease of the cytokines (in all groups) and MMP-3 amount in tissue specimens from gastritis patients. This study did not find a correlation between the IL-1B-31/-511 haplotype frequencies and *H. pylori* infection; however, it might be restricted to the investigated population group [63]. The high levels of both IL-8 and MMP-9 were detected (by ELISA) in the antral biopsies of *H. pylori*–infected individuals with different disease grades. In the IL-8-251 AA genotype Koreans, there was a significant correlation between the MMP-9 level and progression from *H. pylori* infection to chronic atrophic gastritis/intestinal metaplasia and gastric adenocarcinoma [64].

*H. pylori* infection stimulates the migration of leucocytes toward gastric mucosa, and cytokine profiles shape T- and B-cell response. T lymphocytes are represented mainly by pro-inflammatory CD4+ T helper cells, which express IFNγ (interferon gamma), IL-17A or IL-22 and further potentiate the antimicrobial response of epithelial cells, M1 macrophages and other immune cells [65,66]. For example, in gastric cell culture, *H. pylori* and IL-22 synergistically induced MMP-10 via the ERK MAPK pathway. In mice, the epithelial MMP-10 promoted recruitment of CD8+ T cells in gastric mucosa via CXCL16 [37]. Increased level of MMP-10 in gastric mucosa of *H. pylori*-infected patients has been shown to correlate with the gastritis severity [37]. Of note, MMP-10 and some other MMPs are able to shed proteoglycans in the ECM and on the cell surface and release thereby immobilized inflammatory mediators (e.g., IL-8).

IHC has revealed that some MMPs, including MMP-9, were expressed not only in gastric epithelial cells and infiltrated lymphocytes, but also in tissue-resident macrophages in *H. pylori*–infected gastric mucosa [38,40] (Figure 1). Moreover, isolated lamina propria mononuclear cells and blood-derived macrophages produced higher levels of MMP-9 in response to *H. pylori* in vitro [40]. In addition, stroma cells and tissue leucocytes were immunoreactive for MMP regulators TIMP-1, -3 and -4 in biopsies from infected patients [38,67]. Therefore, immune cells contribute to the local disturbances not only via release of cytokines but also through the production of selected MMPs and TIMPs during *H. pylori*–associated gastritis and progression to metaplasia (described also in Section 3.2).

Protection of the gastric mucosa with, for example, rebamipide, has been shown to normalize expression levels of cytokines, ICAM-1, CD44 and overall MMP activity (by zymography), as well as NF-κB activity (by EMSA) in gastric mucosa of *H. pylori* SS1–infected C57BL/6 mice, and to decrease an incidence of chronic atrophic gastritis [68].

### 2.4. MMPs in Ulcer

The development of *H. pylori*–related peptic ulcer is accompanied by the ECM degradation and altered expression and activity of MMPs and their regulators TIMPs. IHC has shown that *H. pylori*–related gastric ulcers express significantly higher levels of MMP-7, MMP-9 and TIMP-1 in comparison to undamaged tissue and NSAID (nonsteroidal anti-inflammatory drug)-related ulcer tissue [69]. The upregulation of MMP-7 in peptic ulcer was related to the cagPAI- and CagA-expressing *H. pylori* [46,70]. The MMP-9 and TIMP-1 levels in ulcers were higher than in the chronic superficial gastritis specimens [41]. Serum levels of MMP-9 (but not MMP-3 and MMP-7) were also higher in patients with *H. pylori*–associated gastric ulcer in comparison to those of patients with *H. pylori*–positive gastritis and cancer [53]. It has been suggested that MMP-9 levels and *H. pylori* infection were risk factors for gastric ulcer recurrence [41]. The expression profile and the role of each MMP in the etiology of the stomach ulcer disease remain to be explored.

## 3. MMPs and Gastric Cancer

### 3.1. MMPs Expression in Tumor

Gastric cancer is the fifth most frequent malignancy worldwide [71]. The Lauren’s histopathological classification subdivides gastric cancer into the intestinal type, diffuse type and mixed/undetermined type [72]. The intestinal and diffuse gastric adenocarcinomas localize in the distal stomach and are related to *H. pylori* infection, but diffuse cancer can be associated with hereditary mutations in, for example, E-cadherin gene. The intestinal histological type is characterized by frequent mutations or nuclear accumulation of β-catenin and strong amplification of c-erbB2/HER2 (human epidermal growth factor receptor 2) [73]. The Correa cascade describes *H. pylori*–associated stepwise development of the intestinal gastric adenocarcinoma [13,15]. MMPs actively impact carcinogenesis and play an undoubtedly important role in the invasion and metastasis in advanced cancer. However, it is difficult to determine the specific impact of each MMP in the process because MMPs have many common substrates, regulate each other and are delivered from different cellular sources in the dynamic tumor microenvironment.

MMP-1 mRNA levels were increased in biopsies from the early gastric distal cancers, especially in *H. pylori*–infected persons [55]. The MMP-1 expression was higher in the intestinal-type gastric cancer than in the diffuse one [55,74]. In about 73% of the cases of advanced cancer, MMP-1 immunoreactivity was associated significantly with peritoneal metastasis and lymph-node metastasis, as well as with worse survival in comparison to MMP-1-negative gastric cancer [75].

Early studies have demonstrated an enhanced expression and activity of MMP-2 and MMP-9 in human gastric carcinoma in comparison to adjacent tissue and have suggested them as prognostic markers of a poor overall survival of patients [76]. However, data concerning a correlation between high MMP-2 and MMP-9 levels and carcinoma types according to the Lauren’s classification were contradictory [74,76]. Later, the MMP-9 expression in gastric tumor has been confirmed to associate with poorly differentiated carcinoma, tumor stage and lymph-node metastasis [77,78]. The RT-PCR data revealed an increased expression of MMP-13, which can be activated by MMP-2 and can activate MMP-9 [4] in gastric carcinoma specimens, in comparison to normal control samples. Increased MMP-13 expression correlated with tumor type (diffuse gastric cancer) and tumor stage in the intestinal gastric carcinomas, but not with *H. pylori* infection [79].

MMP-7, which was found to be upregulated in epithelium in the case of *H. pylori*–associated gastritis, contributes to gastric carcinogenesis [44]. Very early studies demonstrated an enhanced expression of MMP-7 in premalignant gastric lesions and gastric cancer [80,81]. IHC analysis has revealed that MMP-7 was localized predominantly in carcinoma cells but not or weakly in other cell types, and this was especially evident in intestinal-type carcinomas in comparison to diffuse types [81,82]. MMP-7 expression was positively associated with tumor size, invasion and microvessel density and matched the TNM classification [83]. Metastasis in gastric cancer was substantially related to MMP-7 upregulation [82,84,85].

Among MT-MMPs, MT1-MMP is typically expressed in epithelial cells [2]. Nomura et al. [86] have detected that, in about 30% of gastric carcinomas, MT1-MMP was co-localized with MMP-2 on the carcinoma cell surface. In advanced cancer, fibroblasts and vascular endothelial cells were also immune-positive for both MMPs. IHC analysis and ELISA have revealed that MT1-MMP expression was higher in human gastric cancer tissue in comparison to normal gastric and peritoneal tissues. Elevated MT1-MMP was positively associated with tumor invasion, metastasis and poor overall survival of patients [87,88]. In these studies, MT1-MMP expression did not correlate with differentiation status or Lauren’s classification. Inhibition of MT1-MMP in MKN28 cells with siRNA inhibited the activation of pro-MMP-2 and cell invasion in Matrigel [89].

### 3.2. MMPs in Tumor Microenvironment

In *H. pylori*–infected gastric mucosa, MMPs contribute to the inflammatory response via mediating rearrangements in the epithelial layer and lamina. These rearrangements are initially rather protective through facilitating extrusion of damaged cells, wound healing and immune cells’ recruitment to the gastric mucosa. During aggravation of the pathology, MMPs shed cell-surface receptors (e.g., HB-EGF and integrins); degrade collagens and laminins of the basement membrane; and process the latent forms of TGF-β, VEGF (vascular endothelial growth factor), IL-1β, etc., enriched in the ECM [90]. It facilitates excessive infiltration of immune cells, fibroblasts and MSC (mesenchymal stroma cells), leading to further disorganization of tissue and, finally, to the invasion and spreading of transformed cells. The stroma fibroblasts and MSC can also produce MMPs, in particular, MMP-1, MMP-2 and MMP-9 in cell culture, when incubated with the conditioned medium or directly with cancer cells [91,92,93]. Being stimulated by carcinoma cells in the inflammatory environment, stroma and immune cells acquire pro-proliferative properties and support cancer development. TANs (tumor-associated neutrophils), TAMs (tumor-associated macrophages) and CAFs (cancer-associated fibroblasts) in tumor are described to be associated with an unfavorable prognosis and should be explored in the context of gastric cancer therapy [94,95,96,97].

TANs can produce, for example, TNF, IL-8, CCL2/MCP1 and urokinase-type plasminogen activator. IL-17A produced by TANs in vivo and in vitro activates the JAK2 (Janus kinase 2)/STAT (signal transducers and activators of transcription) pathway and expression of the EMT markers vimentin and ZEB (zinc-finger E-box binding protein) 1, a direct transcriptional repressor of E-cadherin and an activator of Snail, Slug and Twist transcriptional repressors. It leads to the enhanced invasion of gastric cancer cells, as shown by using a trans-well system [94].

Local fibroblasts, pericytes and MSC can differentiate toward CAFs [98]. CAFs express, for example, IL-6, IL-8, IL-33, HGF (hepatocyte growth factor), FGF (fibroblast growth factor), TGF-β and CXCL12/SDF-1 [99]. Secretion of IL-33 by CAFs is stimulated by TNF from cancer cells in a NF-κB-IRF-1-dependent manner. In turn, IL-33 activates the ERK1/2-SP1-ZEB2 pathway in cancer cells, and this promotes their migration and invasion [95] (Figure 1).

Interleukins, especially those derived from MSC and CAFs, stimulate the differentiation of macrophages toward tumorigenic M2 phenotype. The M2 macrophages support tumor progression, in part via mediating a T helper cells type-2 immunological response. TAMs express, in particular, IL-6, TGF-β and PD-L1, which suppress T-cell antitumor activity. TAMs-produced MMP-9 activates the PI3K/AKT/Snail signaling in gastric cancer cells and promotes their motility [100], most likely via repressing E-cadherin expression [101].

In addition, all of these cell types, as well as cancer cells, secrete VEGF in response to HIF-1α (hypoxia-inducible factor-1 alpha), which helps to recruit blood vessels and to stimulate further angiogenesis in tumors. Endothelial cells produce, in particular, MMP-1 and MMP-9 in response to angiogenetic factor angiopoetin-2 in the presence of VEGF in co-culture with MKN7 cells. MMPs’ proteolytic activity toward basement membrane surrounding the vessels can favor migration of the endothelial cells [102].

Importantly, tumor-associated cells have the ability to restrict inflammatory response and support tumor growth via suppressing natural killer cells and cytotoxic CD8+ T lymphocytes [66]. Nowadays, bioinformatics tools are applied to analyze the heterogeneous tumor composition. The estimated stromal and immune-cell-type-specific gene signatures in tumor tissue could be used for gastric cancer systematization and outcome prediction [103]. A study by Cao et al. [104] has confirmed a correlation between immunological gene signatures in gastric tumor and survival patterns. The group with high expression in genes specific for hematopoietic stem cells, fibroblasts and endothelial cells and a high stroma score had a worse survival. The group with the best prognosis consisted of genes specific for CD4+ T cells, CD8+ T cells, natural killer cells, macrophages, CD4+ T effector memory cells and T helper cells type 1 [104]. In general, the infiltration of TAMs, CAFs, MSCs and Foxp3+ regulatory T cells and high levels of CXC chemokine receptor 4, CCRs (chemokine C-C motif receptors) 3, 4, 5 and CCR7, HIF-1α, STAT3, COX-2, orphan nuclear receptor 4A2, MMP-2, MMP-7, MMP-9, MMP-21 and macrophage-specific MMP-12 in gastric tumor were associated with poor survival of gastric cancer patients [54,105]. In addition, increased levels of MMP-3, MMP-7 and MMP-11 in serum reflected an advanced stage of gastric cancer and also predicted shorter survival [54,106,107].

Interestingly, several members of the MMP family have been found within tumor-derived extracellular vesicles. Such exosomes can disseminate through the body fluid and provide distant effects, thus participating in cell–cell communication. In vitro, MMP-3-enriched extracellular vesicles from a metastatic murine cancer cell line LuM1 activated the promoter and production of the matricellular protein CTGF (connective tissue growth factor, aka CCN2) in “recipient” cells [108]. This mechanism is unknown for stomach cancer.

### 3.3. Pathways of MMPs Activation in Gastric Cancer

Signaling pathways regulating MMPs expression in tumor cells are represented mainly by the MAPKs and the NF-κB, similar to the *H. pylori*–associated gastritis [55] (Figure 1). MMP-1 secretion can be stimulated by TNF, IL-1β, EGF and histamine through MAPK ERK1/2; MMP-13 is regulated by TNF and IL-1β through p38 MAPK. Moreover, prostaglandins can enhance or inhibit MMPs’ production in cancer cells, context-dependently [109,110]. TNF-induced MMP-9 mRNA and protein expression in SNU216 and SNU668 gastric cancer cells can be suppressed by UO126, a MEK1/2-specific inhibitor [111]. In addition, in MKN45 cells, NF-κB inhibitor PDTC (and a COX-2 inhibitor NS-398) significantly affected *H. pylori*–stimulated MMP-9 and VEGF expression and reduced cellular invasion capacity [112]. In gastric cancer, NF-κB activation can be supported through miRs dysregulation. For example, low miR-7 level is correlated with increased RelA in specimens and poor survival of gastric cancer patients [113]. The delivery of miR-7 abolished the activity of NF-κB; expression of its transcriptional targets, namely ICAM-1, VCAM-1, vimentin, VEGF, MMP-2 and MMP-9; and mitigated gastric cancer metastasis in the lung and liver [113]. The Wnt and STAT3 pathways can be involved in the regulation of MMP-7 expression [114], suggesting that host cellular effectors produced in the tumor microenvironment start playing an important role in MMPs regulation.

MMPs’ production is regulated via histone modifications, by, for example, histone lysine demethylase KDM4B, which co-operates with c-Jun on MMP-1 and IL-8 promoters. Enhanced KDM4B and c-Jun co-expression has been shown to correlate with low survival rate among patients with advanced tumor stage [115]. A histone lysine methyltransferase, SETDB1, which is upregulated in gastric cancer tissue, interacts with ERG (Ets-related gene) transcription regulator to promote the transcription of MMP-9 and cyclin D1 through binding to their promoter regions. In vitro, SETDB1 is activated by *H. pylori* in a transcription factor TCF4-dependent manner; and its overexpression is associated with cell proliferation and metastasis [116] (Figure 1).

The role of polymorphism in MMP genes in the context of a malignant tissue transformation has also been studied. In MMP-1 promoter position-1607, the 1G/2G SNP was investigated because the GGA (in comparison to AGA) variant creates a new Ets-1 binding site. This SNP was not associated with gastric cancer risk, but the 1G/1G genotype was frequent in the intestinal type [117]. Among different MMPs, an MMP-7 gene polymorphism has been found to be associated with *H. pylori* infection status, gastric ulceration, tumor progression and survival of patients [118]. In particular, -181 A/G and G/G genotypes were found more often in gastric tumors of *H. pylori*–positive persons, even though there were no differences in the genotype distribution between gastric cancer patients and controls [119]. Interestingly, the combination of the MMP-7 -181G allele with SNPs in TIMP-1, TGF-β1 or chymase genes in *H. pylori*–infected patients significantly increased the risk of cancer progression and poor survival [119,120,121]. In MMP-9 gene promoter, a -1562 C/T polymorphism has been suggested to be associated with degree of tumor invasion and clinical stage of gastric cancer patients [122,123]. The mechanism and diagnostic significance remain to be clarified.

### 3.4. MMPs and E-Cadherin

During tumor development, a cell-specific phenotype is compromised: epithelial polarity and cell–cell and cell–matrix contacts are disturbed. Furthermore, the cells acquire migratory and invasive properties—a process known as the EMT (Figure 1). Among molecular processes linked to the EMT, the changes in cadherins profile (N-cadherin vs. E-cadherin), expression of vimentin and WNT5A, activation of Wnt/β-catenin signaling pathway and actin cytoskeleton rearrangements play a substantial role [124,125,126]. E-cadherin, a single-pass transmembrane glycoprotein, functions in the adherence junctions mediating cell contacts and, thus, epithelial integrity. In the intestinal epithelial cells, E-cadherin can be cleaved by the MT2-MMP, which interacts through its cytosolic tail with ZO-1 (zonula occludens protein-1) at the apical cell surface. The MT2-MMP-mediated E-cadherin cleavage promotes Src kinase activity in junctional complexes and enhances cell turnover [127]. In gastric cancer cells, *H. pylori*–induced expression of MMP-3 and MMP-7 led to the downregulation of E-cadherin and accelerated cellular migration and invasion. MMPs’ activation was caused by a decrease in miR128 and miR148a levels in the infected SGC-7901 cells [60]. MMP-10 knockout effectively increased the levels of E-cadherin and ZO-1 in *H. pylori*–infected mice when compared to the wild-type mice [37] and affected an invasive phenotype in cell culture [62]. In addition, an elevated expression of MMP-9 and downregulation of E-cadherin have been found to associate with poorly differentiated gastric carcinoma and lymph-node metastasis, and together with increased levels of VEGF and MMP-2, they can serve as malignancy markers in gastric cancer [78,126,128]. The expression of E-cadherin gene can be suppressed through the transcriptional regulators Snail, Slug and ZEB1, which recruit chromatin-modifying proteins to the gene promoter (Figure 1).

## 4. MMPs as Tools and Targets—Concluding Remarks

A lot of novel potential biomarkers have been suggested for screening of the early gastric cancer [129]. The *H. pylori*/CagA antibodies, pepsinogen I level and pepsinogen I/pepsinogen II ratio in human serum seem to be relevant for diagnostics of gastric mucosal atrophy, metaplasia, dysplasia and initial cancer stages [130]. MMPs have also been suggested as biomarkers for gastric cancer, but rather in the advanced stage, taking into account their substantial role in the metastasis. Based on the literature and patients data, Kucera et al. [131] have found that, among MMPs 1–3 and 7–9, systemic levels of MMP-1, MMP-9 and, especially, of MMP-7 can serve as diagnostic markers for gastric cancer; MMP-7 was also evaluated as the best gastric cancer predictor. In addition, a role of *H. pylori*, pepsinogen I and, to a less degree, gastrin as the risk factors in gastric cancer has been confirmed [131]. Thus, the MMPs’ rate reflects disease severity and, when combined with other biomarkers, can be used for diagnostics.

Interestingly, MMPs are being explored as diagnostic tools. NIR (near-infrared) fluorescence probes were created, which are activated intracellularly by MMP-2- or MT1-MMP-dependent proteolysis. These sensors can be used for the determination of MMP activity in cell lines and, probably, for in vivo imaging, taking into account their low autofluorescence and high tissue transparency [132]. Since specific MMPs are increased in primary gastric cancer and metastases, the NIR fluorescent probes can be potentially used for a noninvasive detection of tumors localization and size [88,133].

In addition, MT1-MMP overexpression in some cancers makes this protein a selective target for bicyclic radiolabeled peptides, including DOTA-modified BCY molecules that have been approved in mouse xenograft models, using PET (positron emission tomography) imaging [134]. It remains to be investigated whether such conjugates with therapeutic radionuclides or cytotoxins can be used for targeting MT1-MMP (or another appropriate host protein) in diagnostics and therapy of gastric cancer.

Taking into account an important role of proteolysis in cancer, pharmacological MMP inhibitors were created to improve standard anticancer therapy. The first inhibitory compounds, including marimastat (BB 2516), prinomastat (AG 3340), tanomastat (BAY 12-9566) and batimastat (BB 941), mimicked a substrate or targeted a zinc-binding site in the catalytic domain of protease [135]. Among these firstly developed inhibitors, BAY 12-9566 was the most specific toward MMP-2 > MMP-3 > MMP-9. Despite encouraging results in the experimental and animal studies, the small-molecule MMP inhibitors demonstrated toxicity and a modest effectiveness in clinical trials, which involved rather advanced cancer groups (e.g., marimastat in patients with inoperable gastric cancer) [135]. Afterwards, more specific inhibitors targeting not catalytic (highly homologues) sites but regulatory parts within MMPs were designed, e.g., JNJ0966 toward a region near the cleavage site of pro-MMP-9 and NCS405020 toward the hemopexin domain of MT1-MMP [100,136]. Antibodies REGA-3G12, AB0041 (humanized GS-5745), AB0046 to MMP-9, and LEM-2/15, 9E8 and 3A2 antibodies to MT1-MMP have displayed selectivity and promising results in cell cultures and some mouse disease models [90,137]. In addition, antibody REGA-3G12-drug conjugates have been created to improve antibody effectiveness toward MMP-9 activity [138]. Further progress in protein engineering and computational design has suggested some novel antibody-based inhibitors, in particular, a selective MT1-MMP antibody DX-2400. In breast cancer, for example, DX-2400 blocked MMP activity and pro-MMP-2 processing on tumor and endothelial cells, and it inhibited angiogenesis and tumor dissemination in the MDA-MB-231 xenograft mice model [139]. The new inhibitors still remain to be studied in the context of gastric cancer.

Altogether, extracellular matrix, heterogeneous cell populations and MMPs reciprocally regulate each other. Strong effort has been applied to analyze the proteolysis network in gastric pathology, and some MMPs, e.g., MT1-MMP, came recently in focus as possible diagnostic/therapeutic tools. Further intensive experimental work and computational analysis are required to explore MMPs as specific biomarkers and treatment targets in inflammation and cancer. Moreover, additional studies, probably using new experimental models and inhibitors, are still required to fill a gap concerning distribution, interaction and specific disease-related profiles of the majority of MMPs.

## Data Availability

Not applicable.
